# Impact of charcoal production on soil micronutrients, enzyme activities, microbial composition, and biomass phosphorus in a derived savannah ecosystem of Nigeria

**DOI:** 10.1038/s41598-025-90938-9

**Published:** 2025-09-01

**Authors:** A. J. Adeyemo, I. A. Oluwagbemi, W. O. Ajiboye, E. A. Akinnagbe, T. Y. Akande, M. B. Oyun, M. A. Awodun, D. M. S. Oliveira, D. A. F. Freitas

**Affiliations:** 1 Department of Crop, Soil and Pest Management, Federal University of Technology, Akure, Nigeria; 2 Department of Forestry and Wood Technology, Federal University of Technology, Akure, Nigeria; 3 Department of Soil and Land Resources Management, Federal University, Oye Ekiti, Nigeria; 4 Department of Agricultural Technology, Federal Polytechnic, Ile Oluji, Ondo State Nigeria; 5 Management and Conservation of Soil and Water Laboratory, Federal University of Vicosa, Florestal Campus, Minas Gerais Brazil

**Keywords:** Charcoal production, Deforestation, Microbial composition, P and S enzymes, Soil health, Soil nutrition, Biochemistry, Ecology

## Abstract

Soil functions as the active force managing diverse biogeochemical processes in tropical forest ecosystems, including storing and recycling nutrients and decomposing organic matter. Anthropogenic activities, mainly deforestation on charcoal production, have substantially disrupted these processes, leading to notable changes in microbial activities, enzyme functions, and the availability and soil nutrient status of the derived savannah in southwestern Nigeria. While there is increasing recognition of charcoal’s impact on soil properties, there remains a noticeable research gap in understanding its specific effects on some associated soil microbial properties, soil enzymes, and micronutrients in charcoal production sites. Our investigation assesses soil nutrition, microbial composition, and some selected associated P and S enzymes under charcoal production sites of derived Savanna, Nigeria. Soil samples were systematically collected at 0–15 cm, 15–30 cm, and 30–45 cm in locations associated with charcoal production (CPS) and non-production sites (NPS). The objective was to assess the microbial biomass content in phosphorus and activity levels of microorganisms in soil, focusing on their production of phosphorus and sulfur enzymes, and to examine the overall nutrient release in these diverse environments. The findings revealed Biomass phosphorus (B_p_), Phosphatase (Pho), Thiosulfate dehydrogenase (Tsd), Dimethyl sulfoxide reductase (Dsr), and micronutrients (Mn, Zn, Cu, Co, Fe) were significantly higher in CPS than in NPS. Phytase (Phy) followed a consistent trend at both sites with significant differences among means. Except for copper (Cu), the cobalt (Co), iron (Fe), manganese (Mn), and zinc (Zn) concentrations declined as the soil depth increased in the CPS and NPS across the three locations. This indicates that charcoal production sites in the derived savannah forest of southwestern Nigeria significantly impact soil properties and microbial activities. The higher Bp, Pho, Tsd, and Dsr levels in CPS suggest increased microbial activity and nutrient availability compared to NPS. Additionally, the variation in micronutrient concentrations with soil depth indicates differences in nutrient distribution and availability between the two sites. These findings underscore the importance of further ecosystems to understand the effects of charcoal production on soil ecosystems and to fully develop sustainable management practices that mitigate these impacts.

## Introduction

Agriculture is vital for human survival and as effective as it is, it has led to widespread land degradation due to expanding farming areas. This large-scale change contributes to the depletion of forest ecosystems, carbon sinks that reduce CO_2_ emissions, and other greenhouse gases (GHGs)^1^. Burning or felling of trees, clears a significant portion of the forest, compromising soil health, thereby posing challenges to global food security.

In an ecosystem prone to occasional wildfires, charcoal becomes a common component in soils, as highlighted by studies^2–5^. Moreover, in regions where charcoal production is prevalent, its use has seen a faster growth rate than firewood consumption, emerging as a significantly larger component of overall wood energy consumption in Africa and South America^6^. Despite reported concerns about deforestation and forest degradation linked to charcoal production in many countries^7^, detailed examinations of German soils revealed that charcoal accounted for approximately 45% of the organic carbon content^8^. Conventionally, charcoal generated from most brick kilns is produced in an oxygen-deprived environment, which leads to the production of incomplete combustion waste products such as methane. As a result, the production of charcoal releases greenhouse gases such as carbon dioxide (CO_2_) and methane (CH_4_), which have an impact on global warming.

The burning process in charcoal production has been recognized as a potential factor causing the demise of microorganisms, ultimately leading to a reduction in microbial biomass^9,10^. Investigations report a decline in microbial biomass in the lower layer following the burning process, initiating alterations in the microbiome composition, characterized by a decrease in Fungi and an elevation in Bacteria, specifically actinomycetes^11^. In addition, the microorganism plays a key role in an environment’s micronutrient cycle and solubilizes or mobilizes precipitated nutrient forms to increase the bioavailability of micronutrients^12^. Moreover, by releasing exudates and oxygen, plants alter the composition and diversity of microbial species in the rhizosphere, thus indirectly affecting enzyme activity. Earlier studies have shown that plants exert direct influence on soil enzyme activities by secreting external enzymes. Furthermore, plants indirectly modulate soil enzyme activities by controlling the amount of aboveground litter^13^. The activities of the microorganisms important for Carbon (C ), Nitrogen (N), Phosphorus (P), and Sulphur (S) cycling are also reflected in soil enzymes^14,15^.

In savanna regions, nutrient deficiencies, especially N, P, and S are commonly observed^16^, often attributed to reduced mineralization rates during the rainy season. Rapid nutrient cycling in grassland ecosystems contributes to further soil depletion. Additionally, specific regions may experience micronutrient deficiencies due to soil type or limitations in parent material. Recognizing the spatial and temporal variations in the physiochemical properties of savanna soil is crucial, influenced by factors such as rainfall patterns, slope gradients, vegetation composition, land use practices, and fire frequency^17^. Understanding this diversity is vital for assessing ecosystem functioning, implementing effective land management strategies, and promoting conservation agriculture practices in savanna areas. However, despite the increasing recognition of the substantial influence of charcoal on soil characteristics, there is a noticeable lack of research on its precise effects on microorganisms, microbial biomass phosphorus, soil P and S enzymes, and micronutrients. There are limited or no data on the impact of charcoal production on soil nutrient status, enzyme activity, and microbial communities in this study area. Therefore, this study aims to investigate the influence of charcoal production on some selected micronutrients, enzyme activity, microbial composition, and biomass phosphorus from a derived savannah area in Nigeria.

## Materials and methods

### Experimental site

The study was conducted in three specific locations, as detailed by Adeyemo et al*.*^18^, namely Ìrèle, Òkè-Àkò, and Ìpaò, situated in the Ikole local government area of Ekiti State, Southwestern Nigeria. These locations span from 7° 57′ 22’' N to 5° 32′ 52’' E of Greenwich Meridians and are positioned within the rainforest belt of the tropics. The local climate is characterized by a rainfall range of 120 to 170 mm and temperatures between 24.9 to 27.4 °C. The distance covers about 75.6 kms from Ado Ekiti, the State Capital, to the average heights of about 260 to 300 m above sea level in the uplands^19^. The prevailing soil texture in the study area is sandy clay loam alfisol. Ìrèle, Òkè-Àkò, and Ìpaò are well-known in the Ikole region of Ekiti for their active participation in charcoal production. The soil type is classified as Afisol, is hyperthermic, Oxic, paleustalf, and clayey skeletal, which are found in tropical environments in arid to humid regions beneath hardwood deciduous forest canopy.

### Experimental design and soil sample collection

Soil samples were collected in May 2023, and soil augers and core sampler were used to sample layers (0–15 cm, 15–30 cm, and 30–45 cm) at the three locations and two sites: namely charcoal production sites (CPS) and non-charcoal production sites (NPS). Each combination of CPS and NPS was situated at a distance of only 100 m from each other using a simple random sampling method. One hundred and eight (108) samples were collected from three different locations, totaling 12 sampling points using a manual soil auger equipped with a 20 cm core sampler with an internal diameter of 6 cm for efficient soil sample collection and pulverization. Subsequently, these samples were combined to create 54 treatment combinations, initially structured in 3 × 2 × 3 repetitions, which were later streamlined to three repetitions. Respective soil samples were carefully placed in a polythene bag and labeled with location descriptions for clear identification. Soil samples were air-dried and protected from direct sunlight before being sieved through a 2 mm soil sieve and stored in a cool and ventilated place before laboratory analysis.

### Analysis of soil microorganisms

Total bacteria counts and enumeration was done using the Standard Plate Count Method. Approximately 1 g of each moist soil with an average 20% moisture content was thoroughly mixed with 10 ml of water. One milliliter (1 ml) portion of the samples was pipette in a test tube and serially diluted in another set of test tubes containing 10 ml of sterile distilled water to dilution factor 10^7^. They were plotted on Nutrient agar (NA) for bacterial culture and Potato dextrose agar (PDA) for fungal culture. NA plates were incubated at 37 ◦C for 24 h, while PDA plates were incubated at 25 ◦C for 48 h. Extraction, purification, and enumeration of nematodes was done by careful mixing necessary to avoid harming the organisms during the extraction of Nematodes from newly harvested soils. Sieving of approximately 100 g of soil was used to remove coarse materials, e.g. debris, stones, and roots from the ground. Then, the sieved soil was gently spread into a thin layer on the tissue placed in the sieve and placed on the table. To saturate the soil, 100 ml of water has been carefully introduced into a sieve. By allowing Nematodes to move freely without affecting the water, the tissue acted as a barrier to water contamination. Samples were kept under laboratory conditions for 48 h, with continuous monitoring and additional water to be added if necessary. The sieve was removed after 48 h, allowing water to drain from the soil into the container for about 15 s. The drained water was transferred to the test tube, along with additional washing. The Nematodes were placed in a test tube with approximately 5% chloroform and left to settle for an hour. The supernatant, comprising about 2/3 of the water, was discarded, and the remaining solution was carefully poured into a counting dish. They were examined under a stereomicroscope and counted, and the results were duly reported after allowing the Nematodes to settle for a few minutes. Nematodes were then isolated and identified using a guide by Jonathan^20^ for the most common genera of plant-parasitic Nematodes.

### Analysis of microbial biomass phosphorus

#### Soil sample fumigation method

Ten grams of moist soil was put in a 50 ml beaker, and the beaker was placed in a desiccator. To avoid desiccation of soil samples during fumigation, the desiccator has been lined with wet tissue paper. In the same desiccator, a further 50 ml beaker containing ethanol-free chloroform and boiling chips was added. The desiccator was covered and evacuated by a vacuum pump as the chloroform boiled vigorously for 5 min. Evacuation was repeated 3 times in 15 min to allow the air to pass back into the filtration chamber and facilitate chloroform distribution throughout the soil. Evacuation of the desiccator was performed a fourth time, and 2 min later, the chloroform began to boil very strongly. The valve of the desiccator had been closed, and the desiccator had been placed in the dark for 5 days. Next, another 10 g (un-fumigated) soil was split into two parts, in which KH_2_PO_4_ of a spike of 250 µg was augmented into one part of the non-fumigated samples placed in a 50 ml beaker and placed in a separate desiccator. Before fumigation, this desiccator had been stored in a dark cupboard. After five days of fumigation, the chloroform and the tissue paper were removed and the desiccator was evacuated for 3 min for eight times allow air to pass into the desiccator after each evacuation to remove the chloroform. After 5 days of fumigation, the chloroform and tissue paper were removed and the desiccator was evacuated for 3 min 8 times, allowing air to pass through the desiccator after each evacuation to eliminate the chloroform.

#### Extraction method

The Bray 1 Method^21^ was employed to ascertain microbial biomass phosphorus in the soil. Duplicate samples of 10 g each were measured and placed in centrifuge tubes. Subsequently, 50 ml of Bray 1 solution (0.03 M NH_4_F + 0.025 M HCL) was introduced. The resulting solutions underwent a 5-min shaking period on a mechanical shaker, followed by centrifugation at 2500 rpm for 5 min. The suspensions were then filtered through a No. 42 Whatman filter paper into 50 ml Erlenmeyer flasks. To determine the phosphorus concentration in the extracts, the Murphy and Riley Method was employed. For this, a 1 ml aliquot of the sample was pipetted into a 50 ml volumetric flask, and 8 ml of a solution containing concentrated sulfuric acid, ammonium molybdate, potassium antimony tartrate, and ascorbic acid was added. The solution was filled to the brim of the 50 ml volumetric flask with distilled water. Subsequently, the concentration was determined using a spectrophotometer at a wavelength of 882 nm.

### Soil enzyme activities

#### Phosphorus enzymes

The assessment of phytase (Phy) activities was analyzed according to the ammonium molybdate method used by Sanni et al*.,*^22^, wherein the phosphorus rate was gauged through the elevation of absorbance at 700 nm. In the test tubes, 1 ml of the enzyme solution and 2 ml of the substrate solution were mixed, followed by incubation at 37 °C for 30 min using a controlled Gallenhamp water bath. To halt the reaction, 1 ml of 15% w/v trichloroacetic acid (TCA) was added, and color development ensued with the addition of 1 ml of the colour reagent. To determine soil phosphatase (Pho) activities. About two 2-g portions was weighed from the soil sample and transfer them into screw-cap tubes labeled as “test” and "soil blank." Additionally, another screw-cap tube was designated as the "reagent blank." Following that, 5 ml of a 0.5 M CaCl_2_ solution was pipetted into each of the three tubes, and thorough shaking ensued. For the tubes labeled “test” and "reagent blank," 1 ml of PNPP solution was pipetted, while 1 ml of phosphate buffer was introduced into the “soil blank” tube as a control. After these steps, an incubation period of one hour at 37 °C was instructed for all three tubes. The subsequent actions included transferring 4 ml of the liquid from each tube into labeled 16 × 100 mm test tubes, with caution given to avoid sediment transfer. These test tubes were then subjected to centrifugation for 5 min at 2500 rpm. Following centrifugation, 3 ml of the supernatant was transferred into clean test tubes, with a re-centrifugation step advised if the liquid displayed any cloudiness. Instructions were given to set the spectrophotometer wavelength to 440 nm, adjusting the absorbance to zero using the “soil blank” tube. The absorbance readings for the “test” and “reagent blank” tubes were then to be read and recorded. Additionally, the absorbance was to be set to zero using a blank tube containing 3 ml of CaCl_2_, and the absorbance values for the prepared standards were to be read and recorded. The final step involved plotting the absorbance against concentration to create a standard curve.

#### Sulfur enzymes

The thiosulfate-oxidizing enzyme activities were analyzed using the method described by Trudinger^23^. The standard reaction mixture (1 mI) for measuring thiosulfate dehydrogenase (Tsd) activity contained 25 mM Tris-HC1 (pH 7.5), 1 mM K_3_Fe (CN)_6_, 1 mM Na_2_S_2_O_3_, and enzyme. The measurements, starting with the addition of thiosulfate, were carried out at room temperature. The reduction of ferricyanide was gauged at 420 nm using a spectrophotometer (HP 8524A diode array spectrophotometer), with an extinction coefficient of 1.0 mM 1 cm^-1^. The unit of activity (U) was defined as 1 g/mol of ferricyanide reduced per minute. The unit of activity (U) was defined as 1 g/mol of ferricyanide reduced per minute. Tris–HCl buffer was replaced by 50 mM phosphate buffer for the determination of enzyme activity at reduced pH values. The pH values were adjusted with 0.1 mg NaOH or 0.1 mg H_2_SO_4_, and both before and after enzyme activity were determined. Different amounts of thiosulfate have been introduced into the reaction mixture in kinetic studies. To determine the Dimethyl sulfoxide reductase (Dsr) assay, the procedure involved the using PNPP (p-nitrophenyl phosphate) as a substrate to assess phosphatase activity, while DMSO reductase relied on DMSO (dimethyl sulfoxide) as its specific substrate. Hydrolysis of PNPP resulted in the release of p-nitrophenol, producing a yellow colour that is quantified at 440 nm. In contrast, the reduction of DMSO did not directly produce a coloured product. Phosphate buffer served as a control for phosphatase activity, while for DMSO reductase, various controls were utilized based on the chosen method, such as buffer-only or heat-inactivated enzyme. The calculation of the increase in absorbance over time was used to determine PNPP activity. For DMSO reductase activity, measurement options included the direct reduction in DMSO absorbance at 243 nm or the employment of colorimetric or fluorometric detection in a coupled enzymatic reaction.

### Micronutrients analysis

The soil samples were sieved after being repeatedly crushed with a mortar and pestle. Using a 2 mm soil sieve, the smaller particles were extracted. Two grams of soil samples were weighed and placed in a beaker, and 20 cm^3^ of aqua-regia, and 10 cm^3^ of 30% H_2_O_2_ were added^24^. The addition of H_2_O_2_ was carried out gradually to prevent any potential overflow that would cause material loss. The beakers were covered with a watch glass, and heated for 2 h as the temperature rose to 90 °C. Filters were used to separate the insoluble solid from the supernatant liquid and the volume was increased to 100 cm^3^ using distilled water and the digested sample was analyzed for the presence of heavy metals with the aid of an AAS instrument.

### Statistical analysis

The Minitab 17.0 edition’s general linear model was used to analyze the variance in the acquired data^25^. Data collected on the main and interactive effects of charcoal production sites, depth, and location in measured and selected soil parameters were examined. The PCAs were conducted using the scikit-learn library in Python^26^. The HCA was performed using the scipy.cluster.hierarchy module in Python^27^. Dendrograms were generated to visualize the hierarchical structure and identify optimal clusters. The results were presented in tables and figures, to demonstrate the substantial differences of means obtained at a 5% probability level of confidence, Tukey HSD was used as an experimental post-hoc test. Microsoft Excel 2016 was used to calculate the treatment means and standard errors.

## Results

### Soil physical and chemical properties

The predominant soil type is Alfisol, comprising sand (60.74, 58.95, and 57.42%), clay (28.26, 27.93, and 27.25%), and silt (11.00, 13.11, and 15.33%), with sand being the most abundant, followed by clay and silt. At Ìrèle and Ìpaò, the soil pH was strongly acidic, while it was moderate for Òkè-Àkò. Below the critical value of 10 mg/kg, Ìrèle exhibited the lowest available phosphorus level, whereas Òkè-Àkò and Ìpaò showed significant increases. Nitrogen and potassium (exchangeable) levels exceeded the critical levels of 1 mg/kg and 0.2 cmol/kg, respectively, although calcium and magnesium levels were lower, with the highest values recorded in Òkè-Àkò, significantly differing from the other two locations. The cation exchange capacity (CEC) was lower than the recommended range of between 10–20 cmol/kg, and exchangeable acidity (EA) was higher than the critical limit of 1.0 cmol/kg. At all locations, the base saturation level was slightly above the critical limit of 50% and there were low levels of trace elements including copper, zinc, iron as well as manganese. Our previous work^18^, further presents the detailed physical and chemical properties of soil samples taken from three locations.

### Precipitation and temperature of the research area

The presentation of precipitation and temperature patterns in the study area is outlined in^18^. The cumulative rainfall, measured in millimeters, during the period from April to August 2018 and the recent data in 2023 at the research site is detailed as follows: 120 mm in April, 152 mm in May, 168 mm in June, 170 mm in July, and 131 mm in August. Concurrently, the average temperatures, expressed in degrees Celsius, were recorded as 27.4 °C in April, 26.9 °C in May, 26.2 °C in June, 25.8 °C in July, and 24.9 °C in August.

### The interaction effect of sites and location on phosphorus-sulfur enzymes, and some selected micronutrients concentration

Significant interaction effects between site and location were observed for the activities of phosphorus and sulfur cycling enzymes (Table 1). The recorded interaction effects were significant and varied both in size and direction of their response. Notably, phosphatase (Pho) levels were significantly higher at NPS in Òkè-Àkò and Ìpaò than sulfur enzymes. However, the peak value (2.94 mg/ml/min) was observed at NPS in Òkè-Àkò. Thiosulfate dehydrogenase (Tsd) exhibited significant site-by-location interaction effects, with the highest value recorded at NPS in Ìrèle and the lowest activity level at NPS in Òkè-Àkò, although the variation was insignificant. The recorded interaction effects were both significant and diverse, involving variations in magnitude and direction of response. Additionally, Dimethyl sulfoxide reductase (Dsr) was found to be significantly higher at CPS in Ìpaò, while low activity levels of (0.80 and 0.68 µg/ml/min) were observed at NPS in Òkè-Àkò and Ìpaò, respectively. Lastly, phytase (Phy) activity remained consistent across both sites and depths but showed significant variations among means.

**Table 1:** Interaction effects of charcoal production sites and location on selected micronutrients, P and S enzymes.

Sites	Location	Copper	Colbat	Iron	Manganese	Zinc	(Pho) (mg/ml/min)	Phy	Tsd	Dsr
		(mg/kg)						(µg/ml/min)		
CPS	Ìrèle	0.32^b^	0.10^a^	1.71^c^	0.14^a^	1.60^d^	1.52^d^	0.16^b^	2.67^b^	0.92^d^
	Òkè-Àkò	0.34^a^	0.90^a^	1.30^d^	0.11^a^	1.40^e^	2.94^a^	0.16^c^	3.67^b^	1.03^c^
	Ìpaò	0.31^b^	0.10^a^	2.11^a^	0.11^a^	1.80^a^	1.92^c^	0.16^d^	12.93^a^	1.96^a^
NPS	Ìrèle	0.22^c^	0.10^a^	2.0^b^	0.10^a^	1.80^b^	0.55^e^	0.16^b^	15.22^a^	1.85^b^
	Òkè-Àkò	0.32^b^	0.10^a^	1.20^e^	0.10^a^	1.70^c^	2.45^b^	0.16^a^	1.43^b^	0.80^e^
	Ìpaò	0.31^b^	0.07^b^	1.30^d^	0.20^a^	1.02f.	2.35^b^	0.16^d^	3.81^b^	0.68f.

According to Tukey’s test, means that have the same letter in superscript onacolumn for the same parameterare notdifferent from oneanother (P < 0.05).

Significant interaction effects between site and location were observed regarding the soil nutrient status, as indicated in Table 1. Copper and cobalt were found to be significantly highest at CPS in Òkè-Àkò, however similar trends occurred for Iron in Ìpaò. In contrast, significantly lower values were recorded for cobalt, and zinc in Ìpaò at NPS.

### Interaction effect of sites and depth on phosphorus-sulfur enzymes, and some selected micronutrients concentration

Except for the 30–45 cm soil depth, which displayed lower values (1.78 & 0.84 µg/ml/min) at NPS for Tsd and Dsr, respectively, Table 2 highlights significant interaction effects between site and depth for the selected phosphorus and sulfur enzymes. Particularly noteworthy are the interaction effects observed, such as Tsd showing marginally higher values at the 0–15 and 30–45 cm soil depths at NPS and CPS, respectively, with the highest value recorded at the 0–15 cm soil depth at NPS. Additionally, Pho exhibited significant variation, with the highest activity at the 15–30 cm soil depth at CPS and the lowest activity at the 15–30 cm depth at NPS. Furthermore, at CPS, Cobalt, Iron, and Zinc exhibited their highest concentrations at the 15–30 cm soil depth, while the lowest amount of copper was observed at the 30–45 cm depth. Conversely, at NPS, zinc showed its significantly lowest concentration at the 30–45 cm soil depth. Interestingly, copper was found to be significantly highest at the 30–45 cm soil depth at CPS.

**Table 2:** Interaction effects ofcharcoal production sitesand soildepth on selected soil micronutrients, Pand S enzymes.

Sites	Depth	Copper	Colbat	Iron	Manganese	Zinc	(Pho) (mg/ml/min)	Phy	Tsd	Dsr
		(mg/kg)						(µg/ml/min)		
CPS	0–15	0.31^b^	0.09^b^	1.84^b^	0.10^a^	1.50^c^	2.07^ab^	0.16^c^	4.68^bc^	1.13^d^
	15–30	0.30^b^	0.11^a^	2.0^a^	0.11^a^	1.81^a^	2.31^a^	0.16^c^	4.86^bc^	1.14^c^
	30–45	0.40^a^	0.10^b^	1.30^e^	0.20^a^	1.41^d^	2.00^ab^	0.16^d^	9.73^a^	1.64^a^
NPS	0–15	0.30^b^	0.10^b^	1.52^c^	0.20^a^	1.64^d^	1.82^b^	0.16^a^	10.51^a^	1.35^b^
	15–30	0.30^b^	0.10^b^	1.52^c^	0.11^a^	1.49^c^	1.75^b^	0.16^a^	8.17^ab^	1.14^c^
	30–45	0.30^b^	0.10^b^	1.40^d^	0.10^a^	1.35^e^	1.78^b^	0.16^b^	1.78^c^	0.84^e^

According to Tukey’s test, means that have the same letter in superscript onacolumn for the same parameterare notdifferent from oneanother (P < 0.05).

### Interaction effects of locations and depth on phosphorus-sulphur enzymes, and some selected micronutrients concentration

Significant interaction effects between location and soil depth were noticed in soil P and S enzymes (Table 3). Particularly, at the 0–15 cm soil depth, significantly higher activity levels (3.32 mg/ml/min & 9.41 µg/ml/min) were observed for Pho in Òkè-Àkò and Tsd in Ìrèle, respectively. A similar pattern was observed at the 30–45 cm soil depth, where Ìpaò recorded the highest activity levels for Dsr (1.78 µg/ml/min) and at Ìrèle for Tsd (12.51 µg/ml/min) at 15–30 cm depth. However, at the 30–45 cm depth, notably lower values were observed at Ìrèle for phosphatase and Òkè-Àkò for both Tsd and Dsr activity. Phytase activity remained consistent across the locations and depths but exhibited significant variations among means. In addition, location and depth showed significant interaction effects on the soil micronutrients. For instance, In Ìpaò, Iron, and manganese were significantly highest at the 0–15 cm depth, whereas in Òkè-Àkò, both elements were found in lower amounts at the same depth. At the 15–30 cm depth, copper and cobalt were significantly highest, but they were located in different locations (Ìpaò & Ìrèle) respectively. Zinc exhibited the highest amount at the 0–15 cm soil depth in Ìrèle and the lowest amount at the 30–45 cm depth in both Ìrèle and Òkè-Àkò.

**Table 3:** Interaction effects of charcoal production locationand soil depth on selected micronutrients, Pand S enzymes.

Location	Depth	Copper	Colbat	Iron	Manganese	Zinc	(Pho) (mg/ml/min)	Phy	Tsd	Dsr
	(mg/kg)							(µg/ml/min)		
Ìrèle	0–15	0.28^d^	0.08^cd^	1.93^c^	0.10^a^	2.0^b^	0.59f.	0.16^a^	9.41^ab^	1.60^b^
	15–30	0.24f.	0.12^a^	2.02^b^	0.11^a^	1.71^c^	1.50^de^	0.16^a^	12.51^a^	1.41^c^
	30–45	0.28^de^	0.10^a-c^	1.60 f.	0.20^a^	1.30^a^	1.01^ef^	o.16^a^	4.93^bc^	1.15^e^
Òkè-Àkò	0–15	0.38^ab^	0.06^d^	0.94^i^	0.09^a^	1.41^e^	3.32^a^	0.16^b^	4.51^bc^	1.11f.
	15–30	0.26^ef^	0.11^ab^	1.70^d^	0.13^a^	1.71^c^	2.55^b^	0.16^b^	1.92^c^	0.85^h^
	30–45	0.35^c^	0.11^a^	1.10^h^	0.11	1.30^g^	2.21^bc^	0.16^c^	1.21^c^	0.78^i^
Ìpaò	0–15	0.24 f.	0.09^bc^	2.17^a^	0.30^a^	1.33f.	1.92^cd^	0.16^d^	8.86^ab^	1.00^g^
	15–30	0.40a	0.08c^d^	1.61 ^e^	0.10^a^	1.50d	2.04^bc^	0.16^d^	5.12^bc^	1.17^d^
	30–45	0.37^bc^	0.08^cd^	1.33^g^	0.11^a^	1.40^e^	2.45^b^	0.15^c^	11.13^a^	1.78^a^

According to Tukey’s test, means that have the same letter in superscript onacolumn for the same parameterare notdifferent from oneanother (P < 0.05).

### Interaction effects of charcoal production sites by location by soil depth on Phosphorus-sulphur enzymes, and soil selected micronutrients concentartion

At CPS, Pho, Tsd, and Dsr activity increased across all locations with increasing soil depth, except in Òkè-Àkò where it decreased (Table 4). Phy showed a consistent trend across all locations and depths at both CPS and NPS. For soil nutrients, Fe decreased across locations and depths. However, Co and Zn followed similar trends, except in Òkè-Àkò. Cu and Mn activity increased across locations with increasing soil depth, but Mn showed no significant difference. At NPS, Cu, Co, and Fe increased across two locations with increasing depth, except for Ìpaò. Mn and Zn decreased down the soil profile at all locations, except in Òkè-Àkò. Pho, Tsd, and Dsr decreased with increasing soil depth, except Pho, which increased at Ìpaò across soil depth.

**Table 4:** Interaction effect of charcoal production sites by locationby soil depth on selected micronutrients, P and S enzymes.

	Copper	Colbat	Iron	Manganese	Zinc	(Pho) (mg/ml/min)	Phy	Tsd	Dsr
	(mg/kg)						(µg/ml/min)		
Site (S)									
CPS	0.32^a^	0.10^a^	1.70^a^	0.13^a^	1.57^a^	2.13^a^	0.16^a^	6.82^a^	1.30^a^
NPS	0.29^b^	0.09^b^	1.48^b^	0.12^a^	1.50^b^	1.78^b^	0.16^a^	6.42^a^	1.11^b^
Location (L)									
Ìrèle	0.27^b^	0.10^a^	1.84^a^	0.12^a^	1.66^a^	1.03^c^	0.16^a^	8.95^a^	1.39^a^
Òkè-Àkò	0.33^a^	0.10^a^	1.23^c^	0.11^a^	1.53^b^	2.69^a^	0.16^a^	2.55^b^	1.32^b^
Ìpaò	0.33^a^	0.83^b^	1.70^b^	0.16^a^	1.41^c^	2.13^b^	0.16^a^	8.37^a^	0.91^c^
Soildepth (SD)									
0–15cm	0.30^b^	0.08^b^	1.70^b^	0.15^a^	1.60^a^	1.94^a^	0.16^a^	7.60^a^	1.24^a^
15–30cm	0.30^b^	0.10^a^	1.80^a^	0.12^a^	1.50^b^	2.03^a^	0.16^a^	6.51^a^	1.24^a^
30–45cm	0.33^a^	0.10a	1.33^c^	0.11^a^	1.60^a^	1.89^a^	0.15^b^	5.76^a^	1.14^b^
Interactions									
S x L	*	*	*	ns	*	*	*	*	*
S x SD	*	*	*	ns	*	*	*	*	*
L x SD	*	*	*	ns	*	*	*	*	*
S x L x SD	*	*	*	ns	*	*	*	*	*

According to Tukey’s test, this means having the same letter in superscript onacolumn for the same parameterare notdifferent from oneanother (P < 0.05).

### Interaction effects of charcoal production sites by location by soil depth on phosphorus-sulphur enzymes, and some selected micronutrients

Three-way analysis of variance presented in [Table 5], indicates the effect of charcoal production on some selected P and S enzymes at the CPS and NPS. Regardless of location and soil depth, there were no significant differences (*P* > 0.05) indicated in Phy and Tsd between CPS and NPS, but higher and significant differences (*P* < 0.05) were recorded in CPS with Pho and Dsr. The same table also showed the effect on some selected P and S enzymes at different locations. Irrespective of the production sites and soil depth, there were significant differences in soil P and S status amongst the three different locations. Phosphatase was significantly (*P* < 0.05) higher in Oke-Ako than in Irele and Ipao, higher significant differences were also recorded in Tsd and Dsr at Irele. Although no statistically detectable differences were indicated amongst the three locations. There were significant differences in soil P and S enzyme status as affected by soil depth (Table 4). Phytase and Dsr decreased in the order of increasing soil depth with the lowest value recorded in 30 – 45 cm depth, however, Tsd and Pho with no significant differences recorded higher values in 0–15 and 15 – 30 cm depths respectively. There were significant interaction effects of site by location by soil depth recorded in P and S enzymes. The effect of charcoal production on some soil nutrient status at the CPS and NPS [Table 5], showed that Cu, Co, Fe, Mg, and Zn were higher in CPS than NPS. In terms of location, Cu, Co, and Mn were found to be higher in Ìpaò compared to the other two locations. However, Fe and Zn were found to be higher at Ìrèle with significant differences (*P* > 0.05). Furthermore, Cu and Co increased with increasing depth while Fe and Mn followed the opposite trend. Finally, Zn remained consistent across the soil profile showing a significant difference (*P* > 0.05).

**Table 5:** Correlation among variables.

	Zn	Mn	Fe	Co	Cu	Dsr µg/ml/min	Tsd µg/ml/min	Phy µg/ml/min	Pho mg/ml/min	Nematode (10^3^)	Fungi (10^5^)	Bacteria(10^8^)
	(mg/kg)											
Mn	− 0.14ns											
Fe	0.09 ns	0.06 ns										
Co	0.21 ∗ ∗	− 0.10 ∗ ∗	0.40 ∗ ∗									
Cu	0.05 ns	− 0.16 ∗ ∗	− 0.69 ns	− 0.37 ∗ ∗								
Dsr	0.46 ∗ ∗	− 0.07 ∗	0.31 ∗ ∗	− 0.24 ∗	− 0.24 ∗ ∗							
Tsd	0.40 ∗ ∗	0.10 ns	0.36 ∗ ∗	− 0.17 ns	− 0.41 ∗	0.87 ∗ ∗						
Phy	0.18 ns	− 0.10 ∗ ∗	− 0.01 ns	0.24 ∗ ∗	− 0.36 ∗ ∗	− 0.04ns	− 0.05 ∗					
Pho	− 0.37 ns	0.05 ∗	− 0.53 ∗	− 0.17 ns	0.39ns	− 0.27 ∗	− 0.28 ∗ ∗	− 0.28 ∗ ∗				
Nematode	− 0.06 ∗	− 0.040 ns	− 0.28 ns	− 0.08 ns	− 0.10 ∗ ∗	− 0.15 ∗ ∗	0.04ns	0.40 ∗ ∗	− 0.07 ns			
Fungi	0.11 ∗ ∗	0.18 ∗ ∗	− 0.09 ∗ ∗	− 0.08 ns	0.11 ∗ ∗	0.09ns	0.21 ∗ ∗	− 0.15 ns	0.28 ∗	0.11 ∗ ∗		
Bacteria	0.03 ns	− 0.15 ns	− 0.01 ns	0.23 ∗ ∗	0.09ns	− 0.36 ∗	− 0.45 ∗ ∗	0.26 ∗ ∗	0.20 ns	− 0.04 ns	0.04ns	
Biomass P	− 0.06 ns	− 0.04 ns	0.20 ∗ ∗	0.10 ∗ ∗	− 0.06 ∗	− 0.19 ∗ ∗	− 0.22 ∗ ∗	0.09 ns	0.19 ∗ ∗	− 0.06 ∗ ∗	0.01 ∗	0.63 ∗ ∗

Variables in pairs exhibitinga ( +)correlationand havinga P- value < 0.05 tend to increase together. In pairs witha (-)correlationanda P-value ˂ 0.05, one variable tends todecreaseas the other increases. For P-value > 0.05, no significant relationship is observedbetween the two variables. The presence of one star ( ∗) signifies that thecorrelation is significantat the 0.05 level; two stars (∗ ∗) indicate significanceat the 0.01 level.

### Correlation (r) among microbial biomass P, microorganisms, phosphorus-sulhur enzymes, and some selected micronutrients concentration

The correlation analysis revealed significant associations among various parameters in the study area as presented in [Table 5]. The pairwise Pearson correlations indicate the significance of these associations. A weak negative correlations was observed between Mn and Zn, as well as Fe and Zn, indicating that fluctuations in Mn and Fe levels are not consistently linked to changes in Zn levels. Similarly, there is a weak positive correlation between Co and Zn, and Cu and Zn, suggesting that variations in Co and Cu levels are not significantly tied to changes in Zn levels. Furthermore, there are weak negative correlations between Nematode (10^3^) and Zn, and Biomass P and Zn, indicating that variations in Nematode (10^3^) and Biomass P levels do not consistently correspond to changes in Zn levels. Conversely, a strong positive correlation is detected between Dsr and Zn, and Tsd and Zn, both of which are statistically significant. This implies that as levels of Dsr and Tsd increase, Zn levels tend to increase.

Transitioning to correlations between different elements, a very weak positive correlation between Fe and Mn was observed, while weak negative correlations exist between Co and Mn, Cu and Mn, Nematode (10^3^) and Mn, Fungi (10^7^) and Mn, Bacteria (10^8^) and Mn, Biomass p and Mn, Dsr and Mn, Tsd and Mn, Phy and Mn, Pho and Mn. None of these correlations are statistically significant, indicating that variations in these elements are not reliably associated with changes in Mn levels. Also, a moderate positive correlation was identified between Co and Fe, and strong negative and moderate negative correlations are noted between Cu and Fe, and Nematode (10^3^) and Fe, respectively. These correlations are statistically significant, suggesting that as Co levels increase, Fe levels tend to increase, while an increase in Cu and a change in Nematode (10^3^) levels are linked to a decrease in Fe levels.

The correlations between Fe and Fungi (10^7^), Bacteria (10^8^), and Biomass P are very weak and not statistically significant. However, a moderate positive correlation is found between Dsr and Fe, and a strong negative correlation is observed between Pho and Fe, both of which are statistically significant. These results indicate that as Dsr levels increase, Fe levels tend to increase, while an increase in Phosphatase (Pho) levels is associated with a decrease in Fe levels. The analysis extends to correlations between elements such as Co, Cu, Nematode (10^3^), Fungi (10^7^), Bacteria (10^8^), and Biomass P, revealing various weak and very weak correlations, none of which are statistically significant. Shifting to Tsd and Fe, a moderate positive correlation is noted, indicating that as Tsd levels increase, Fe levels tend to increase. When exploring relationships with Cu, there is a moderate negative correlation with Co, a very weak negative correlation with Nematode (10^3^), Fungi (10^7^), and Biomass p, and a weak positive correlation with Bacteria (10^8^). While the correlation with Nematode (10^3^) is not statistically significant, correlations with Co and Fungi (10^7^) are, suggesting that as Cu levels increase, Co levels tend to decrease, and an increase in Cu levels is associated with a decrease in Fungi (10^7^) levels. Bacteria (10^8^) exhibits very weak correlations with Nematode (10^3^), Fungi (10^7^), and Biomass P, none of which are statistically significant. Moving on to Phy, there is a weak positive correlation with Co, a very weak positive correlation with Fungi (10^7^), and a very weak negative correlation with Nematode (10^3^). None of these correlations are statistically significant, suggesting that variations in Phy levels are not reliably associated with changes in Co, Fungi (10^7^), or Nematode (10^3^) levels. Phosphatase displays strong positive correlations with Cu, a strong negative correlation with Fe, and a weak positive correlation with Fungi (10^7^), all of which are statistically significant. These findings indicate that as Pho levels increase, Cu levels tend to increase, Fe levels tend to decrease, and there is a weak positive association with Fungi (10^7^) levels.

The analysis concludes with correlations involving Biomass P, showing weak positive correlations with Co and Tsd, and a marginal positive correlation with Phy. None of these correlations are statistically significant, suggesting that variations in Biomass P levels are not reliably associated with changes in Co, Thiosulfate dehydrogenase, or Phy levels. Finally, the correlations between various enzymes Dsr, Tsd, Phy, and Pho are explored, revealing weak and very weak correlations, with only the correlation between Tsd and Dsr being marginally statistically significant.

The correlation coefficients and their empirical coupling regression equations between the amounts of micronutrients including enzymes and increasing soil depths at the charcoal production sites (CPS) and non-charcoal production sites (NPS) are summarized in Table 6. Copper and cobalt were found to be positively correlated with increasing soil depths at both sites in contradistinction to iron and manganese which were negatively correlated with increasing soil depths. Zinc was negatively and positively correlated with increasing soil depths at the charcoal production site and non-charcoal production site, respectively. There was a highly significant correlation between the amounts of Pho and increasing soil depth at both sites. The correlation between Phy and soil depth at both sites was practically zero. Both Tsd and Dsr were positively correlated with increasing soil depth at the charcoal production site, while the reverse is the case at the non-charcoal production site. On the contrary, no significant relationship was found for Phytase (Phy) for both CPS and NPS. A simple linear correlation and regression analysis between enzymes, micronutrients (Y), and increasing rates of soil depth (X) showed significant positive and negative relationships (Table 7). In Ìrèle, regressing enzyme and micronutrient parameters (Y) against increasing soil depth (X) of Ìrèle indicated highly significant (P ≤ 0.001) negative relationships for Tsd, Dsr, Fe, and Zn. In addition, a highly significant positive relationship was found for Pho, Co, and Mn. Moreover, Cu showed a negative relationship with a correlation coefficient of 0. Furthermore, the regressing enzyme and micronutrient parameters (Y) against increasing soil depth (X) of Òkè-Àkò indicated highly significant (P ≤ 0.001) positive relationships for Phy, Dsr, Co, Fe, and Mn whereas a negative relationship found for Pho, Tsd, Cu, and Zn. Lastly, for Ìpaò, all four enzymes showed a highly significant positive relationship likewise all but Co, Fe, and Mn showed a negative linear correlation and regression relationship against increasing soil depth (Table 7).

**Table 6:** Linear correlation and regression between micronutrientsand enzymes (*Y*) and increasing soil depth (*X*) for charcoal production site (CPS)and non-charcoal production site.

Soil parameters	Site			
	CPS		NPS	
	Correlationcoefficient (r)	Regression equation	Correlationcoefficient (r)	Regression equation
Copper	0.817	Y = 0.2467 + 0.00300 X	1.00	Y = 0.3000 + 0.000667 X
Cobalt	0.500	Y = 0.0900 + 0.000333 X	1.00	Y = 0.1000 + 0.000667 X
Iron	− 0.736	Y = 2.253–0.0180 X	− 0.866	Y = 1.6000–0.00400 X
Manganese	− 0.908	Y = 0.0367–0.00333 X	− 0.908	Y = 0.2367 – 0.00333 X
Zinc	− 0.214	Y = 1.663–0.0030 X	0.999	Y = 1.78333 + 0.009667 X
Pho	− 0.223	Y = 2.193–0.0023 X	− 0.569	Y = 1.8233–0.00133 X
Phy	0.0	Y = 0.00 + 0.0000X	0.0	Y = 0.00 + 0.00 X
Tsd	0.881	Y = 1.37 + 0.1683 X	− 0.967	Y = 15.55–0.2910 X
Dsr	0.874	Y = 0.793 + 0.01700 X	− 0.995	Y = 1.6200–0.01700 X

**Table 7:** Linear correlationand regression between micronutrients and enzymes (*Y*) and increasing soil depth (*X*) at different locations.

Soil parameters	Location					
	Ìrèle		Òkè-Àkò		Ìpaò	
	Correlation coefficient	Regression equation	Correlation coefficient	Regression equation	Correlation coefficient	Regression equation
Copper	− 0.0	Y = 0.2667 + 0.00000 X	− 0.240	Y = 0.360—0.00100 X	0.764	Y = 0.207 + 0.00433 X
Cobalt	0.50	Y = 0.0800 + 0.00067 X	0.866	Y = 0.0432 + 0.001667 X	− 0.866	Y = 0.09333—0.000333 X
Iron	− 0.746	Y = 2.180—0.01100 X	0.199	Y = 1.087 + 0.0053 X	− 0.982	Y = 2.543—0.02800 X
Manganese	0.908	Y = 0.0367 + 0.00333 X	0.500	Y = 0.0900 + 0.00067 X	− 0.843	Y = 0.360—0.00633 X
Zinc	− 0.995	Y = 2.3700—0.02333 X	− 0.259	Y = 1.583—0.0037 X	0.409	Y = 1.340 + 0.00233 X
Pho	0.461	Y = 0.613 + 0.0140 X	− 0.976	Y = 3.803—0.03700 X	0.954	Y = 1.607 + 0.01767X
Phy	0.561	Y = 0.1593 + 0.000300 X	1.00	Y = 0.1500 + 0.0006670 X	0.941	Y = 0.15367 + 0.000533 X
Tsd	− 0.588	Y = 13.43—0.149 X	− 0.950	Y = 5.85—0.1100 X	0.374	Y = 6.10 + 0.076 X
Dsr	− 0.996	Y = 1.8367—0.01500 X	0.949	Y = 1.24 + 0.01100 X	0.950	Y = 0.537 + 0.02600 X

### Selected soil microbial activities

**Fig. 1:**
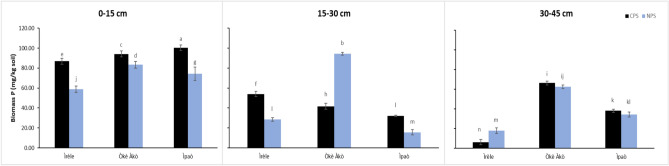
Microbial biomass phosphorus in the soils of CPS and NPS in different locations and depths in Ikole, Ekiti, Southwestern Nigeria. Vertical bars indicate standard errors of the means (n = 5). Bars with same alphabet (s) within a soil layer for the same parameter are not significantly different (P < 0.05).

**Fig. 2:**
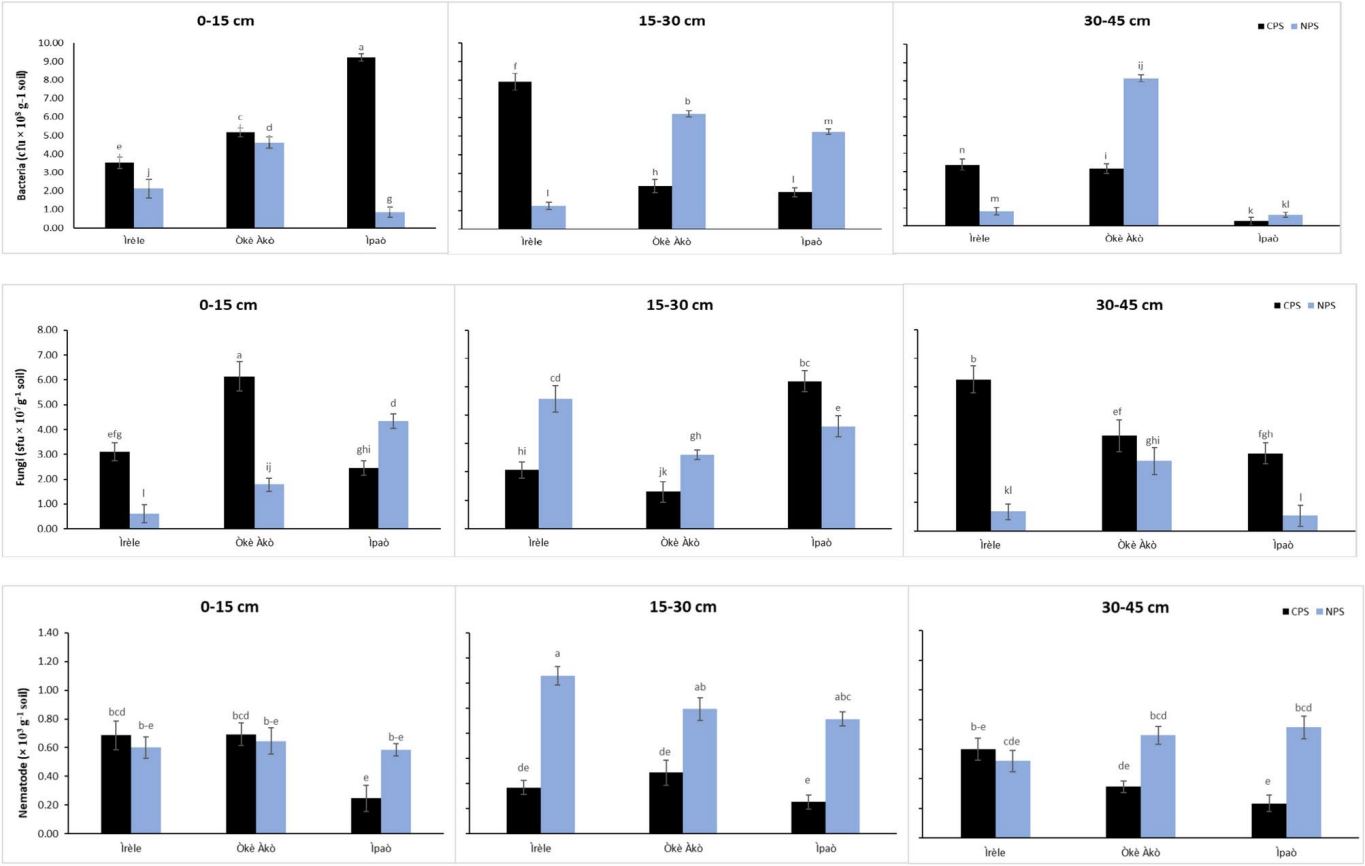
Bacterial, Fungi, and Nematodes counts in the soils of CPS and NPS in different locations and depths in Ikole, Ekiti, Southwestern Nigeria. Vertical bars indicate standard errors of the means (n = 5). Bars with the same alphabet (s) within a soil layer for the same parameter are not significantly different (P < 0.05).

The biomass phosphorus content in the designated areas exhibited its highest levels at CPS in the 0–15 cm soil depth in Ìrèle, Òkè-Àkò, and Ìpaò Fig. 1. Subsequently, it gradually declined as the soil depth increased across the profile. Conversely, at NPS, the biomass phosphorus content demonstrated higher patterns at the 0–15 cm depth and reached its peak at the 15–30 cm soil depth in Òkè-Àkò. Then it further decreased with increasing depth across the soil profile. In the study area, the bacteria activity displayed a significant increase in the 0–15 cm soil depth, followed by a gradual decline across the three locations Fig. 2. Specifically within the NPS, Òkè-Àkò and Ìpaò exhibited significantly higher bacteria activity at a depth of 15–30 cm. At the 30–45 cm soil depth, Òkè-Àkò registered the highest bacteria activity, while Ìpaò showed the lowest. Conversely, at CPS, bacteria were relatively abundant at the 0–15 cm depth in all three locations and at the 15–30 cm depth in Ìrèle; thereafter, it progressively decreased with an increase in depth. Fungal activity was found to be abundant at CPS across various soil depths and in all three locations Fig. 2. Furthermore, it was observed to intensify with increasing soil depth at CPS. It was significantly higher at 0–15 cm in Ìpaò, at 30–45 cm in Ìrèle, and reached its peak at 0–15 cm soil depth in Òkè-Àkò. Conversely, at NPS, Fungi exhibited a different pattern, with a significant increase at the 15–30 cm soil depth across the three locations. Additionally, at the 30–45 cm depth, fungal activity was notably lower at Ìrèle and Ìpaò. 4Nematode abundance was found to be significantly higher at natural production sites (NPS) compared to charcoal production sites (CPS) in soil layers of 0–15 cm, 15–30 cm, and 30–45 cm across all three locations Fig. 2 . However, there was less variation in nematode abundance at the 0–15 cm soil depth, unlike Ìrèle, where the highest abundance was observed at the 15–30 cm soil depth at NPS. Similar patterns were observed in Òkè-Àkò and Ìpaò.

#### Principal component and hierarchical cluster analyses

**Fig. 3:**
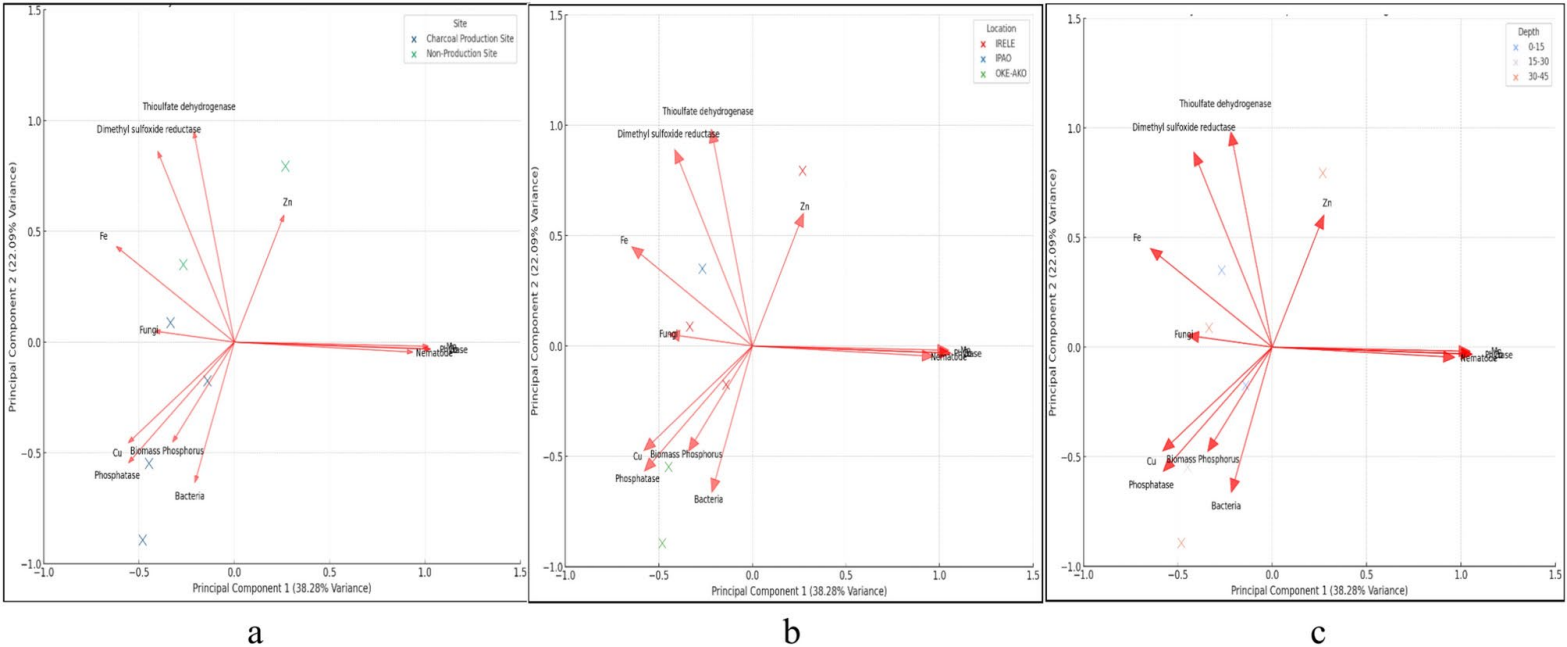
Biplots obtained by PCA under charcoal production sites in Irele, Ipao, and Oke-Ako between the depths of 0–15, 15–30, and 30–45 cm. (**a**) Mbp, Bac, Fun, and Nem; (**b**) Phd, Phy, Tsd, and Drs; (**c**) Cu, Co, Fe, Mn and Zn.

Principal Component Analysis (PCA) analysed the relationship between a number of biological, chemical and enzymatic variables at charcoal production sites (Fig. 3). The PCA variables include biomass, bacterial and fungal activities, nematode population, phosphatase and phytase activities, sulfur cycle enzyme activities including thiosulfate dehydrogenase and dimethyl sulfoxide reductase as well as heavy metals such as Cu, Co, Fe, Mn and Zn. The first principal component, PC1, which accounted for 38.28% of the total variance, was mainly related to variables which are related to general biological activity for instance microbial biomass P and bacterial activity. This component may therefore be a proxy for the site nutrient status and general biological activity and thus areas with higher values of these variables will be more biologically active and richer in nutrients.

The second principal component (PC2) for 22.09% of the variance represented various ecological factors mainly affected by nematode activity and fungal distribution. This shows that there is a combination of different factors that work together or in contrast with each other to determine the soil ecosystem, as represented by the directions of their loadings. This can be to show balances or conflicts that are occurring within the ecosystem for instance competition for resources (Fig. 3a). The first principal component was mainly related to biological production and the dynamics of nutrients. The areas with high biomass and bacterial activity for instance Irele had high PC1 scores indicating that they had nutrient rich and biologically active soils. The second principal component was mostly affected by biophysical parameters including nematodes and fungi.

Places like Ipao showed opposite scores for PC2, which might be related to the soil’s microbial balance or other environmental stresses. Oke-Ako had lower scores on PC1 and PC2, reflecting degraded or nutrient-poor conditions, probably as an influence of lower microbial and enzymatic activities (Fig. 3b). The same plot also showed clear clustering associated with shallower depths of 0–15 cm (Fig. 3c). The depths positioned high with PC1 scores are strongly interrelated with higher biomass P, bacterial activity, and enzymatic contribution, referring to nutrient-rich and biologically active soils. The intermediate zone between the extremes in both factors PC1 and PC2 was dominated by depths within the 15–30 cm range; it expresses moderate biological productivity and enzymatic activity. While the higher soil depth between 30–45 cm showed lower scores on PC1 and a moderate score on PC2, these depth levels recorded reduced biological activities and nutrient cycling, probably as a result of a reduction in microbial populations and availability of organic matter.

**Fig. 4:**
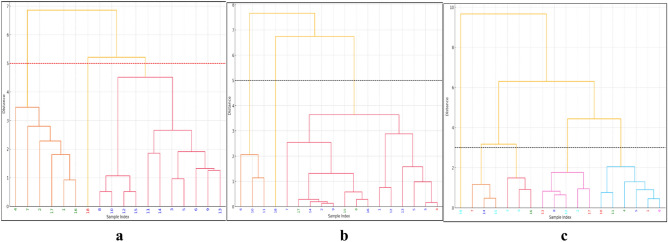
Dendogram obtained by HCA under charcoal production sites in Irele, Ipao, and Oke-Ako between the depths of 0–15, 15–30, and 30–45 cm. (**a**) Mbp, Bac, Fun, and Nem; (**b**) Phd, Phy, Tsd, and Drs; (**c**) Cu, Co, Fe, Mn and Zn. The index number is recorded in Table Y above.

Clusters are colour-coded, with samples from Cluster One marked in green, Cluster Two in blue, and Cluster Three in red. The red dotted line at a distance of 5 represents the threshold line, which is the level at which the dendrogram has been cut to define these three clusters. This display gives an overview of the grouping of the samples and how each cluster is defined based on the hierarchical analysis. Samples index 1, 2, 4, 7, 16, and 17 in Table 8 belong to Cluster One; 3, 5, 6, 8, 9, 10, 11, 12, 13, 14, and 15 to Cluster Two; and Cluster Three contains sample 18 only (Fig. 4a). This representation spreads the samples over three clusters and hence provides a more orderly overview of their falling in a group based on the analysis. Cluster One is dominated by high microbial biomass P and bacterial counts, indicating a biologically active environment with probably a good level of nutrient supply. High fungal activities further support ecological functions such as decomposition and nutrient cycling in the cluster. Although moderate, nematode activities point toward a healthy soil ecosystem which could be potentially beneficial for plant growth and soil structure. Cluster Two shows a more moderate level of microbial biomass P and bacterial count, probably reflecting less nutrient-rich or disturbed soils than in Cluster One. The fungal activities still support certain ecological roles, but the general ambient conditions may be less than perfect for strong microbial activities to take place. The rate of nematode activities remains similar to that of Cluster One, showing balanced ecological interactions. Cluster Three has only one sample with notably lower microbial biomass P and fungal activities, which may indicate a probably stressed environment with limited ecological functions. Higher nematode activity could be indicative of stress responses or dominance of certain nematode groups that may be detrimental or indicative of conditions that need further investigation. Thus, Cluster One is the richest and most biologically active, hosting thriving ecosystems; Cluster Two represents more moderate ecological conditions, possibly requiring some management to enhance biological activity. Cluster Three may need detailed investigation on account of its potential environmental stress or unique conditions.

**Table 8:** Interaction effect of sites by locations by depths, showing the sample index on the Hierarchical clustering dendrograms in charcoal production sites of derived savanna, Nigeria.

Sample index	Sites	Location	Depth
1	CPS	Irele	0–15
2	CPS	Irele	15–30
3	CPS	Irele	30–45
4	CPS	Ipao	0–15
5	CPS	Ipao	15–30
6	CPS	Ipao	30–45
7	CPS	Oke-Ako	0–15
8	CPS	Oke-Ako	15–30
9	CPS	Oke-Ako	30–45
10	NPS	Irele	0–15
11	NPS	Irele	15–30
12	NPS	Irele	30–45
13	NPS	Ipao	0–15
14	NPS	Ipao	15–30
15	NPS	Ipao	30–45
16	NPS	Oke-Ako	0–15
17	NPS	Oke-Ako	15–30
18	NPS	Oke-Ako	30–45

CPS:charcoal production sites, NPS: Non-production sites.

Figure 4b Provides HCA of enzyme activities. Colour-coding of the samples is identical as in Fig. 4a. Cluster One includes samples 6, 10 and 11. These samples are grouped together because of their similar level of at least one of the assayed enzymatic activities and may thus perform similar biochemical functions in similar environmental conditions. Samples 1, 2, 3, 4, 5, 7, 8, 9, 12, 13, 14, 15, 16, 17 were grouped in Cluster Two. This is the biggest cluster, indicating general similarity across a wide range of samples, possibly reflecting a common environmental background or similar metabolic activity level. At the same time, Cluster Three contained only one sample of 18. It stands alone in its cluster, reflecting unique enzymatic activity profiles that are considerably different from all other samples. Such could indicate either an outlier condition or a different environmental or physiological state. These clusters divide the samples according to their enzymatic activities and allow drawing some conclusions about ecological or biological similarities and differences. Cluster One had moderate phosphatase but very high thiosulfate dehydrogenase activity, suggesting a strongly sulfur-cycling-capable environment that may point toward enrichment in either sulfur or organic matter. The high levels of dimethyl sulfoxide reductase possibly suggest adaptation to oxidative stresses or environments with a high organic sulfur compound (Fig. 4b). The largest cluster, Cluster Two, exhibits high phosphatase activity, suggesting nutrient-rich conditions that are favourable for phosphorus cycling, important to biological growth and soil health. Lower levels of thiosulfate dehydrogenase and dimethyl sulfoxide reductase suggest less specialization in sulfur compound metabolism and may represent a more typical terrestrial or soil environment with high moisture content. Cluster Three contains one outlier sample that has very low phosphatase and thiosulfate dehydrogenase activities but very high phytase activities. Such a profile may indicate a special ecological niche or physiological state where phosphorus is cycled in a peculiar way, possibly in waterlogged soil or in a specialized microbial community focused on organic phosphorus mobilization.

For the micronutrients, Cluster One represents samples 3, 7, 9, 14, 15, and 16 (Fig. 4c). Samples which could potentially show similarities regarding micronutrient concentration within the studied areas fall within this cluster. Cluster Two consists of samples 1, 2, 4, 5, 6, 8, 10, 11, 12, 13, and 17. Since it is the largest cluster, these samples share more similarities across the measured micronutrients concentrations and may indicate that these samples have a common environmental or geological background. In Cluster Three, only one sample, 18, was found to stand alone in its cluster, indicating a uniqueness of micronutrient concentration profiles which are significantly different from other samples. This could imply another kind of environmental or contamination scenario. The cluster also showed a high and moderate level of the following metals: copper, iron, and manganese. It might suggest samples pertaining to areas influenced either by natural mineral deposits or those places with activities relating to charcoal production in moderation. For example, iron and manganese are common in both natural deposits and as industrial byproducts through combustion and mining. Cluster Two is the largest cluster and tends to have generally lower values of Cobalt and Zinc compared to the other clusters. These samples are probably representative of typical background levels from less contaminated situations. The low metal concentration suggests a minimal influence due to charcoal production or less exposure to pollution sources. Cluster Three is dominated by extremely high levels of Cobalt and Manganese, far above the other clusters. This cluster indicates a special environmental setting, possibly near mining sites or areas with geological anomalies. High levels of Cobalt and Manganese can be linked to specific types of mineral deposits or industrial contamination.

## Discussion

Soil nutrition is essential for sustaining plant growth and ecosystem functions. Microorganisms play a vital role in recycling organic matter (OM) and nutrients in soil. They act as repositories during the immobilization and as providers during the mineralization of labile nutrients, as highlighted by Ribeiro et al*.,*^28^. In soils prone to leaching, immobilization is a significant mechanism for retaining nutrients. Phosphorus (P) is often scarce in terrestrial ecosystems, relying on efficient recycling mechanisms from biomass, particularly evident in mature ecosystems^29–31^. Despite the challenge of P availability, soil microorganisms significantly contribute to forest P nutrition by mobilizing and immobilizing P^32^, thereby influencing plant P nutrition through their biomass composition^33^. Similar to the patterns seen in phosphorus dynamics, the rise in soil nitrate-nitrogen levels in charcoal-enriched surface soils may be due to reduced leaching or improved biological cycling of nitrogen, as suggested by Cayuela et al*.,*^34^. As a result, increased nitrogen and phosphorus levels could have a positive effect on the plant community in forests, similar to what has been observed in grassland systems^35,36^.

### Microbial contribution to phosphorus in charcoal soil

Phosphate-solubilizing microorganisms, which rely on organic matter inputs, produce chelating organic acids to unlock phosphate bound to minerals^37^. As organic matter diminishes and microbial biomass decreases, phosphates and other nutrients are released into the soil. Indigenous microorganisms can transform insoluble phosphates into soluble forms under favorable conditions, underscoring their importance in P cycling. Acid phosphatase, which is actively released by both tree roots and microbial cells, plays a key role in phosphorus cycling and is affected by soil microclimate, as well as the presence of organic carbon and phosphorus, as noted by Pang et al.,^38^. As stated in the result the bacteria activity displayed a significant increase in the 0–15 cm soil depth, followed by a gradual decline across the three locations. This suggests that the population of bacteria tends to rise after a fire due to the increased availability of carbon sources. McCormack^39^ predicted a decrease in fungal abundance and an increase in bacterial abundance after the application of charcoal, which raises the pH. This increase in pH favored bacterial populations over fungal populations, as observed by Rousk et al*.,*^40^. Fungal activity was found to be abundant at CPS across various soil depths and in all three locations. This result is supported by studies by Iftikhar^37^ and Neuberger^41^ which have demonstrated that the application of charcoal promotes the colonization of arbuscular mycorrhizal fungi in agricultural plants. Also, temperatures exceeding 50 °C lead to the death of heat-sensitive microbes, with fungi being more susceptible than bacteria. Interestingly, nematode abundance was found to be significantly higher at non-charcoal production sites (NPS) compared to charcoal production sites (CPS) in soil layers. These findings suggest that the addition of charcoal could lead to changes in soil properties, resulting in reduced nematode abundances and changes in feeding type composition at kiln sites^42^. The distribution of nematode feeding types indicates that charcoal addition promotes fungi over bacteria within the litter microbial community, although bacteria still dominate. More studies have also shown that the addition of biochar can suppress infestations of plant-parasitic nematodes in soils with elevated levels of nitrogen and phosphorus^43^. The higher soil acidity and the introduction of magnesium, calcium, potassium, and manganese in charcoal soils may influence the nematode community.

### Enzyme activities and nutrient dynamics in charcoal soils

Phosphorus enzyme activities, particularly phosphatase and phytase, were significantly higher in charcoal production sites (CPS) compared to non-charcoal production sites (NPS). Sulfur enzyme thiosulfate dehydrogenase also exhibited higher activity in CPS, indicating enhanced nutrient dynamics in charcoal-amended soils. A study conducted by^44^ noted that using biochar, a form of charcoal, led to heightened activity of thiosulfate reductase within soil samples. The researchers attributed this augmentation in activity to microbial communities within the biochar capable of generating sulfur enzymes. Similarly, in research conducted by^45^, it was observed that the application of charcoal resulted in increased activity of dimethyl sulfoxide reductase within soils. The authors proposed that this enhancement in activity stemmed from the charcoal’s ability to create a conducive environment for microbial communities producing sulfur enzymes.

Additionally, certain micronutrient elements such as Cu, Co, Mg, Zn, and Fe showed varied distribution patterns between CPS and NPS soils, highlighting the influence of charcoal on soil nutrient dynamics. The adsorption properties of charcoal hold these nutrients and prevent further leaching. Nonetheless, the influence of charcoal production on soil micronutrients is linked to the elevated temperatures characteristic of the process, which modify the chemical and physical properties of the soil. According to a study by Ghezzehei et al*.,*^46^, charcoal production alters the soil’s pH, nutrient content, and water-holding capacity, affecting the soil micronutrient levels. Additionally, the process may lead to soil compaction, which further reduces the soil’s ability to retain micronutrients. More so, Fagbenro et al.,^47^ stated that the volatilization of essential nutrients such as nitrogen, sulfur, and potassium contributes to soil nutrient loss through burning wood for charcoal production. The study has also shown that soil pH levels are usually changed and this leads to acidity in the soil.

Micronutrients including iron, manganese, zinc, and copper all necessary for plant growth are less readily available in locations where charcoal is produced due to the high levels of acidity in the soil^48^. According to Matson^49^, soil acidity is a major factor affecting micronutrient availability in soils in the tropics. Micronutrients are essential elements required in small quantities for plant growth and development. These include iron, zinc, copper, and manganese, among others. The availability and uptake of micronutrients in soil are influenced by various factors, which include soil texture, pH, and organic matter content. Furthermore, the impact of charcoal production on soil micronutrients includes the depletion of soil organic matter. Also, the burning of wood for charcoal decreases soil organic matter, leading to soil degradation and diminished soil nutrient retention capacity.

The profile distribution of micronutrients and enzymes in soil displayed variations with increasing soil depth. Generally, the highest micronutrient concentration has always been observed to occur at surface levels^50^. However, further analysis reveals variation in depth for different micronutrients depending on the environmental factors. For example, at CPS, the positive linear correlation and regression between Cu, Co, Phy, Tsd, and Dsr with increasing depth implies increased availability or activity levels. But in a case where the relationship is negative, this means that as depth increases the parameters decrease in value. For instance, Fe, Mn, Pho, Tsd, and Dsr all displayed a negative relationship at NPS. This result agrees with the findings of^48^, stating that as depth increased down the profile micronutrients such as Mn, Zn, and Fe decrease in concentration. It is widely understood that enzyme activity typically diminishes as soil depth increases^51^. Piotrowska-Długosz et al.^52^ provided further insight, indicating that hydrolytic enzyme activity (FDAH) consistently decreases with depth, whereas oxidative enzymes exhibit a different pattern, sometimes showing higher activity in deeper soil layers compared to the upper ones. This reduction in enzyme activity is linked to various factors, including pH changes, air–water conditions, and other variables^53^.

### PCA and HCA

Results of the PCA highlighted the intricate role that both biological and chemical variables played in defining ecological characteristics in charcoal production and non-production sites. Analysis gave a nuanced look at ecosystem dynamics that distinguished variable contributions such as biomass P, bacteria, fungi, nematodes, and micronutrients in Fig. 3a. The large influence of biomass P and bacteria on PC1 gives evidence of the critical role microbial processes play in ecosystem productivity and nutrient cycling, as noted by^54^. These findings agree with several studies showing that microbial biomass is a key indicator of soil fertility and environmental quality, such as^55^. The contrasting impacts of fungi and nematodes on PC2 suggest complex interactions within the soil ecosystem, which may involve competition for resources or different roles in plant and soil health^56^. This aligns with research demonstrating these organisms’ dual roles in supporting and challenging plant growth depending on environmental conditions^57^. Directional vectors of micronutrients indicate the pollution issues that are not addressed and may lead to ecological degradation in the future^55,57^ Thus, management strategies should be focused on mitigating charcoal production and enhancing soil management practices to reduce micronutrients that could lead to soil pollution due to increased concentration in these ecosystems^56^.

The PCA further underlines different ecological dynamics across the studied locations by showing trends corresponding to known environmental processes and site management practices. In this respect, the significant loading of microbial biomass P and bacterial activity on PC1 could account for differences among locations; thus, locations like Irele are dominated by nutrient-rich soils with strong microbial ecosystems (Fig. 3b). The present data align with studies emphasizing microbial activities’ role in enhancing soil fertility and ecosystem productivity, according to^59^, and^58^. The contrasting contributions of fungi and nematodes to PC2 highlight the intricate balance within soil ecosystems. High fungal activity may indicate decomposition-driven nutrient cycling, while nematode populations often reflect soil health and stress conditions^56,60^. Locations with higher PC2 scores, such as Ipao, suggest a predominance of one group over the other, potentially due to site-specific environmental conditions^57^. This distribution is reflected in the variable vectors of micronutrients like Cu, Fe, and Zn in the PCA shown in Fig. 3b.

Sites with high micronutrient concentrations, such as Oke-Ako, may thus be associated with geochemical variations-a finding that agrees with those from^55^. These support that trace elements in soil environments have a dual function: being nutrients and possible stressors to the ecosystem. Phosphatase and phytase, contributing to the phosphorus cycle through hydrolysis of organic phosphorus, were strongly positively related to PC1. These enzymes are crucial in improving the availability of phosphorus in the soil, which is rather limiting, especially in resource-poor ecosystems, as was evidenced in some plots at Ipao. As reported, phosphatase activity indicates phosphorus availability and soil biological activity, and this has been in agreement with a number of studies, for instance^63^, and^62^. The contributions to PC1 and PC2 were also from thiosulfate dehydrogenase and dimethyl sulfoxide reductase, which participate in sulfur metabolism.

Locations like Oke Ako, with significant sulfur-related enzymatic activity, may reflect unique geochemical conditions or microbial adaptations. Sulfur metabolism enzymes play a role in soil redox processes and influence microbial community dynamics ^63,64^]. The variable contributions of micronutrients (Cu, Co, Fe, Mn, and Zn) highlight the impact of geochemical and anthropogenic factors on soil health. With higher concentrations of micronutrients, Oke-Ako may be subjected to environmental stress or contamination. Though essential for biological processes, excessive metal concentrations inhibit enzymatic activity and microbial growth, impacting soil functionality^57^.

The strong influence of biomass and bacterial activity on PC1 emphasizes the importance of biological productivity in soil depth differentiation. Shallower depths, like 0–15 cm, showed higher biological activities (Fig. 3c), due to more availability of organic matter and better conditions for microbial growth^59,58^]. Large contributions from phosphatase and phytase toward PC1 point toward the prime role of enzymes in phosphorus availability of the upper soil layers. In general, such results are in agreement with studies indicating that enzymatic activities in nutrient cycling and soil fertility are very crucial^61,62^.

Similarly, sulfur-related enzymes such as thiosulfate dehydrogenase and dimethyl sulfoxide reductase contributed to PC1 and PC2, reflecting their involvement in sulfur transformations and microbial redox processes in the deeper layers^63,64^. The contrasting contributions of nematodes and fungi to PC2 illustrate the dynamic balance of soil health indicators across depths. Intermediate and deeper layers, dominated by the activity of nematodes, indicate stress-adapted soil ecosystems with lower microbial diversity or nutrient cycling efficiency. According to^56^, the micronutrients Cu, Fe, and Zn influenced the PCA components, showing moderate concentrations in deeper soil depths. While these micronutrients are very important for microbial and enzymatic functions, too high levels may act as a stress factor and inhibit biological processes^57^.

Cluster one shows high microbial biomass P which often signifies good soil health, normally because microbes are very imperative in the nutrient cycling and decomposition of organic matter that forms structure in the soil. Increased bacterial counts imply increased availability of nutrients, especially N and P, which are some of the most vital elements that plants need to grow, as outlined by^65^. The association of fungal activity with complex organic material breakdown contributes to enhanced soil fertility and structure^66^. In Cluster Two, moderate microbial and fungal activities suggest a rather stable but less active ecosystem compared to Cluster One (Fig. 4a). This may be due to the previous charcoal production, inherent soil properties, or recent disturbances in the study area^67^. Nematode activity similar to Cluster One suggests a balanced soil food web, crucial for maintaining soil function and health^68^. Low microbial and fungal activities in Cluster Three suggest poor soil health or a stressed environment, potentially due to pollution, overuse, or lack of organic matter^69^. High nematode activity might indicate beneficial or harmful nematode populations^70^.

Figure 4b shows the three clusters identified from the enzymatic activity data for phosphatase, phytase, thiosulfate dehydrogenase, and dimethyl sulfoxide reductase. Cluster One is characterized by very high thiosulfate dehydrogenase activity, suggesting excellent sulfur metabolism abilities. Sulfur metabolism plays an important role in different ecological and industrial processes, including the degradation of pollutants and nutrient cycling. Other studies, such as by^71^, have discussed microbial sulfur metabolism for its ecological roles and biotechnological applications and pointed out the importance of sulfur-oxidizing and -reducing bacteria in environmental sulfur cycles. Cluster Two shows high phosphatase activity, usually associated with nutrient-rich environments that are conducive for biological growth.

The most crucial processes in biological systems concern the cycling of phosphorus, and phosphatases play a great role herein. Recent findings have been able to underline the importance of phosphatase enzymes in hydrolyzing organic phosphorus compounds, thereby releasing phosphate for plant growth and soil microbial functions, as underlined by^72^. Cluster Three was exceptionally high in phytase activity, combined with very low activities of the other measured enzymes, which suggested a unique specialization or stress adaptation. Phytase is important in breaking down phytate, the principal form of phosphorus in many soils and otherwise inaccessible to most organisms. It points to the role of the enzyme in enhancing phosphorus bioavailability within both environmental and agricultural environments. A review by^73^ discusses phytases regarding soil phosphorus management and plant nutrition.

Figure 4c presents the three clusters as derived from the concentration of micronutrients, Copper, Cobalt, Iron, Manganese, and Zinc, in the study areas. Cluster One presents samples with moderate to high concentrations of metals such as copper, iron, and manganese. Recent findings by^74^ indicate that environmental pathways for these metals are crucially influenced by natural processes and human activities. The book elaborates on how different metals migrate through ecosystems, underlining natural abundance and human influence, both acting in a dual manner to develop metal concentration profiles of soils. Generally, Cluster Two is characterized by low concentrations of Cobalt and Zinc. It probably represents background levels typical of less contaminated environments; thus, these areas are not seriously affected by charcoal production. Adriano^75^ discussed the natural biogeochemical cycles of trace elements in 'Trace Elements in Terrestrial Environments, underlining that the baseline soil concentration is critical for distinguishing between the natural and anthropogenic contributions. Zhang et al. 2022^76^ expand on those recent developments concerning methodologies and findings related to trace element cycling. The text provides a comparative study of the concentration of metals in various environments, important in defining anomalous values that can indicate contamination. Cluster Three is dominated by extremely high levels of Cobalt and Manganese, by far higher than in other clusters. This indicates a peculiar setting, probably close to mining activities or places with special geological formations rich in these metals. As pointed out by^59^, certain metals are concentrated in particular regions due to either the local geology or other intense human activities. This book explicitly talks about how metals like cobalt and manganese may accumulate in soils, including the environmental health risks resulting from their high concentrations near production sites. Recent works, such as that by^77^, confirm these findings and extend the mechanisms responsible for such distributions.

## Conclusion

The study findings reveal significant differences in the abundance of micronutrients, P and S enzymes, and microbial activity between charcoal production sites and natural production sites. Specifically, charcoal production sites showed higher levels of micronutrients (Co, Cu, Fe, and Zn), enzymes Pho, Tsd, Dsr and Phy, and microbial activity compared to natural sites, indicating a strong influence of the production site on their distribution. Interestingly, Mn levels remained consistent across both types of sites, suggesting a minimal impact of the production site on its distribution. Copper and Fe exhibited variations based on location, while Co and Mn showed little variation by location, with the highest quantities found in Ìpaò. Zinc displayed a clear decreasing trend across the three locations, indicating significant differences among means. These findings underscore the role of local factors or unique production practices in shaping the distribution of these elements across different locations. Furthermore, soil microbial populations play a crucial role in regulating soil carbon storage, nutrient cycling, and overall soil health. Additionally, soil microbial communities can influence nitrogen cycling and fixation, leading to increased plant productivity. Given the large and diverse nature of microbial communities inhabiting the soil environment, microbial indices have been used earlier as a tool for observing soil quality. Microorganisms respond quickly to changes in soil environmental conditions, as such microbial indices can be used to monitor soil health and investigate the impact of environmental factors, nutrient addition, or some soil parameters on microbial communities. Therefore, soil nutrition, microbial composition, and enzyme activities play integral roles in sustaining ecosystem functions, particularly under charcoal production sites in derived savanna ecosystems. Understanding these dynamics is crucial for sustainable land management practices and ecosystem health.

## Supplementary Information

Supplementary Information.

## Data Availability

The data that support the findings of this study are available from Dr. Adebayo Jonathan ADEYEMO but restrictions apply to the availability of these data, which were used under license for the current study, and so are not publicly available. Data are however available from the authors upon reasonable request and with permission of Dr. Adebayo Jonathan Adeyemo.
