# Supplementary material for: Impact of charcoal production on soil micronutrients, enzyme activities, microbial composition, and biomass phosphorus in a derived savannah ecosystem of Nigeria
Source: Sci Rep. 2025 Sep 1;15:32101. doi: 10.1038/s41598-025-90938-9 (PMC12402133; doi:10.1038/s41598-025-90938-9)
Supplement: Supplementary file 1 — Supplementary Information. [file 41598_2025_90938_MOESM1_ESM.docx]

**Soil nutrition, microbial composition and some selected associated P n S enzymes under charcoal production sites of derived Savanna, Nigeria.**

Adeyemo, A. J^1,3^; Oluwagbemi, I. A^1^; Ajiboye W.O^1^; Akinnagbe, E.A^1,4^; Akande, T. Y^3^; Oyun M.B^2^; Awodun M.A^1^.

1. Department of Soil, Crop and Pest Management, Federal University of Technology, Akure, Nigeria.
2. Department of Forestry and Wood Technology, Federal University of Technology, Akure, Nigeria.
3. Department of Soil and Land Resources Management, Federal University Oye Ekiti, Nigeria
4. Department of Agricultural Technology, Federal Polytechnic Ile-Oluji, Nigeria

**Charcoal production effect on selected micronutrients, P and S enzymes and interaction between production site, locations and soil depth**

|  | Location | Depth | Copper Cobalt Iron Manganese Zinc    .......................(mg/kg) ................................. | | | | | | (Pho)  (mg/ml/min) | Phy Tsd Dsr  ................(µg/ml/min) ................ | | | | | |
| --- | --- | --- | --- | --- | --- | --- | --- | --- | --- | --- | --- | --- | --- | --- | --- |
|  |  | | | | |  | | | | |  |  | | **(mg/ml/** |  |
| CPS | Ìrèle | 0-15 | 0.31^d-f^ | 0.11^ab^ | 2.03^c^ | | 0.10^a^ | 1.84^a^ | 0.78^ef^ | 0.16^b^ | | | 0.47^d^ | | 0.70^jk^ |
|  |  | 15-30 | 0.30^e-g^ | 0.12^a^ | 2.10^b^ | | 0.11^a^ | 1.30^a^ | 2.06^bc^ | 0.16^b^ | | | 1.04^d^ | | 0.76^i^ |
|  |  | 30-45 | 0.40^bc^ | 0.08^bc^ | 1.01^j^ | | 0.22^a^ | 1.60^h^ | 1.71^cd^ | 0.16^b^ | | | 6.50^cd^ | | 1.31^f^ |
|  | Òkè-Àkò | 0-15 | 0.31^b-d^ | 0.05^d^ | 1.08^i^ | | 0.10^a^ | 1.04^k^ | 3.86^a^ | 0.16^c^ | | | 7.07^cd^ | | 1.37^e^ |
|  |  | 15-30 | 0.23^hi^ | 0.11^ab^ | 1.80^f^ | | 0.13^a^ | 0.96^m^ | 2.80^b^ | 0.16^c^ | | | 3.37^cd^ | | 0.99^g^ |
|  |  | 30-45 | 0.50^a^ | 0.11^ab^ | 1.00^j^ | | 0.12^a^ | 2.11^b^ | 2.15^bc^ | 0.15^f^ | | | 0.57^d^ | | 0.71^j^ |
|  | Ìpaò | 0-15 | 0.30^fg^ | 0.10^ac^ | 2.42^a^ | | 0.12^a^ | 1.63^g^ | 1.56^c-e^ | 0.15^e^ | | | 6.50^cd^ | | 1.31^f^ |
|  |  | 15-30 | 0.23^hi^ | 0.11^ab^ | 2.12^b^ | | 0.10^a^ | 2.02^c^ | 2.06^bc^ | 0.15^e^ | | | 10.17^c^ | | 1.68^d^ |
|  |  | 30-45 | 0.31^ce^ | 0.08^de^ | 1.81^ef^ | | 0.12^a^ | 1.74^f^ | 2.15^bc^ | 0.16^d^ | | | 22.12^a^ | | 2.88^a^ |
| NPS | Ìrèle | 0-15 | 0.30^gh^ | 0.05^d^ | 1.84^e^ | | 0.11^a^ | 2.11^b^ | 0.40^f^ | 0.16^b^ | | | 18.35^ab^ | | 2.50^b^ |
|  |  | 15-30 | 0.20^i^ | 0.11^ab^ | 1.94^d^ | | 0.11^a^ | 2.16^a^ | 0.93^d-f^ | 0.16^b^ | | | 23.97^a^ | | 2.05^c^ |
|  |  | 30-45 | 0.20^i^ | 0.11^ab^ | 2.11^b^ | | 0.09^a^ | 1.01^kl^ | 0.31^f^ | 0.16^b^ | | | 3.37^cd^ | | 0.99^g^ |
|  | Òkè-Àkò | 0-15 | 0.42^a^ | 0.07^cd^ | 0.80^l^ | | 0.08^a^ | 1.77^e^ | 2.77^b^ | 0.16^a^ | | | 1.95^d^ | | 0.85^h^ |
|  |  | 15-30 | 0.30^e-g^ | 0.12^a^ | 1.53^g^ | | 0.13^a^ | 1.32^i^ | 2.30^bc^ | 0.16^a^ | | | 0.47^d^ | | 0.70^jk^ |
|  |  | 30-45 | 0.30^gh^ | 0.12^a^ | 1.22^h^ | | 0.10^a^ | 2.00^c^ | 2.27^bc^ | 0.16^a^ | | | 1.85^d^ | | 0.84^h^ |
|  | Ìpaò | 0-15 | 0.20^i^ | 0.08^bd^ | 1.93^d^ | | 0.40^a^ | 1.03^k^ | 2.27^bc^ | 0.16^d^ | | | 11.23^bc^ | | 0.69^k^ |
|  |  | 15-30 | 0.44^a^ | 0.05^d^ | 1.20^i^ | | 0.10^a^ | 1.00^lm^ | 2.02^bc^ | 0.16^d^ | | | 0.07^d^ | | 0.66^m^ |
|  |  | 30-45 | 0.42 | 0.07^cd^ | 0.90^k^ | | 0.10^a^ | 1.04^k^ | 2.74^b^ | 0.15^f^ | | | 0.13^d^ | | 0.67^l^ |

According to Tukey's test, means that have the same letter in superscript on a column for the same parameter are not different from one another (P < 0.05).
